# Does patient’s position count during Endoscopic Retrograde Cholangio-pancreatography? Left lateral decubitus versus prone position

**DOI:** 10.12669/pjms.39.5.6932

**Published:** 2023

**Authors:** Laima Alam, Rao Saad Ali Khan, Farrukh Saeed, Farrukh Sher, Rao Zaid Ali Khan

**Affiliations:** 1 Laima Alam, FCPS, MRCP, ESEGH, CHPE, Consultant Gastroenterology. Bahria International Hospital, Rawalpindi, Pakistan; 2 Rao Saad Ali Khan, FCPS Med, FCPS Gastro, FRCP, Consultant Gastroenterology and Transplant Hepatologist. Pak Emirates Military Hospital, Rawalpindi Pakistan; 3 Farrukh Saeed, FCPS Med, FCPS Gastro, Head of Department of Gastroenterology. Pak Emirates Military Hospital, Rawalpindi Pakistan; 4 Farrukh Sher, FCPS Med, Fellow Gastroenterology, Pak Emirates Military Hospital, Rawalpindi Pakistan; 5 Rao Zaid Ali Khan, Research Assistant. Pak Emirates Military Hospital, Rawalpindi Pakistan

**Keywords:** Endoscopic retrograde cholangio-pancreaticography, Left lateral decubitus, outcomes, Post-ERCP pancreatitis, Prone position

## Abstract

**Objective:**

To compare the efficacy and safety of left lateral decubitus versus prone position during endoscopic retrograde cholangio-pancreaticography (ERCP).

**Methods:**

This prospective single-centre cohort study was carried out at Pak Emirates Military Hospital from January to June 2021. Patients requiring ERCP were subsequently allotted LL or PP group randomly (unequal randomization) except patients with recent abdominal surgery, in-dwelling catheters, raised intra-abdominal pressure, cervical spine abnormalities and limb contractures. Qualitative data was analysed using frequencies and chi square statistics whereas, quantitative data was analysed using mean±SD and student T or Mann Whitney U-test.

**Results:**

A total of 114 patients were enrolled according to the inclusion criteria with 62(54%) males and majority of the patients (42%) belonging to the age group 31-45 years. The most common ERCP indication was choledocholithiasis (36%). Technical success was achieved in 109(96%) patients with no statistically significant difference between the two groups. The total time of procedure, time for deep cannulation, time for acquiring therapeutic goal and ERCP complexity level were all similar between the two groups. The rate of inadvertent PD cannulation and PEP were relatively higher for the PP group but were statistically non-significant through univariate and logistic regression analyses and the only outcome measure that showed significance was multiple cannulations in the PP group.

**Conclusion:**

The study concludes that LL is non-inferior to PP and both positions have comparable outcomes with non-significant differences in terms of technical success rate, complications (specifically PEP), total procedure time, time required for deep cannulation and attainment of goal, ERCP complexity level and inadvertent PD cannulation.

## INTRODUCTION

Although a widely used pancreatico-biliary procedure world-wide, endoscopic retrograde cholangio-pancreaticography (ERCP) is nevertheless associated with considerable complications and mortality in the absence of due regard to the quality indicators.^1^ Large volumes of research is available on the risk factors for complications (intra and post ERCP procedures) that can be narrowed down to two most relevant components, endoscopist’s expertise and the ease of cannulation.^2^

Traditionally, ERCP has been performed in the prone position (PP) and has been linked to greater facilitation of deep biliary cannulation and superiority of fluoroscopic images of the pancreatico-biliary tree.^3^ Left lateral decubitus position (LL), on the other hand, is preferred by the anaesthetists and is considered relatively comfortable for the patients with limited cervical mobility, morbid obesity, abdominal distension, recent abdominal surgery and raised intra-abdominal pressure, limb contractures and pregnancy.^4^ Some of the setbacks quoted in the literature regarding LL are poor opacification of the proximal biliary tree, scope torsion and the need of the endoscopist to look away from the monitors during cannulation.^3^,^4^ Supine position (SP), however, is rarely tried in the endoscopy suite due to its potential risk of cardiopulmonary collapse and a relatively low technical success rate.^5^

A thorough literature review revealed that efficacy and safety of ERCP in PP versus SP has been reported in randomized controlled trials but there is a paucity of research regarding LL position. No such study was found locally by using multiple search engines like PakMediNet, Google Scholar and PubMed. This prospective study was designed to compare the efficacy and safety of prone versus left lateral decubitus position during ERCP in terms of technical success rate, time during cannulation and goal attainment, inadvertent PD cannulation and complications.

## METHODS

This study was designed as a prospective single-centre cohort study carried out at Pak Emirates Military Hospital, Rawalpindi from January 2021 to June 2021 after obtaining ethical committee review (EC/406/2022, dated January 2021) and patients’ consent.

### Inclusion and Exclusion Criteria

All patients, age 15 years and above with indication for ERCP and consenting to the study were enrolled. Exclusion criteria included active shock and hemodynamic instability, coagulopathy, severe cardio-pulmonary disease and pregnancy. The patients underwent unequal allocation randomization to either of the two groups; prone (PP) or left lateral (LL). The exceptions to randomization were patients with morbid obesity, tense ascites, recent abdominal surgery, in-dwelling catheters, raised intra-abdominal pressure, cervical spine abnormalities and limb contractures.^6^ These were assigned LL group to prevent discomfort and obvious complications.

The study was performed in a dedicated tertiary care advance GI procedure suite by two high volume Consultant Endoscopists with 95% success rate of biliary cannulation and 400 ERCPs per year. Sedation with intravenous midazolam (0.05-0.1 mg/kg) and propofol (0.5-1 mg/kg) was provided to all patients. All patients were provided with oxygen at 2L/minutes through nasal cannula and were continuously monitored for BP, pulse, oxygen saturation, ECG and level of sedation. A strict cannulation protocol of a total of five cannulation attempts with no more than two inadvertent PD cannulations was followed. If deep cannulation was not achieved through this standard method, double guidewire and/or pancreatic stent assisted procedure, use of early Needle Knife Sphincterotomy (NKS), NKS as a last resort or TPS and/or combined sphincterotomy were tried according to the indication of the procedure and the local anatomy. Both the operators used the same technique for deep cannulation and the demographics, extensive cannulation data, laboratory and outcome measures were noted for every patient. All patients received pre-procedure rectal indomethacin, were observed for 6 hours post-ERCP and those with significant abdominal pain were retained for 24 hours.^7^

### Definitions

Cannulation attempt was defined as a continuous contact between the papilla and the sphincterotome for at least five seconds.^7^ Resorting to NKS after a total of five attempts at cannulation with standard method was defined as early NKS. In some of the cases, NKS was tried earlier at the endoscopist’s discretion considering the local anatomy. Difficult biliary cannulation was defined as more than five attempts at papillary cannulation, more than five minutes spent to cannulate after papilla was clearly visualized or more than one inadvertent PD cannulation.^8^

Technical success was defined as free and deep instrumentation of the biliary tree.^7^ Total time of the procedure was defined as the time from initial intubation to the procedure termination.^9^ Pancreatitis was defined as abdominal pain and more than three times rise in serum amylase levels 24 hours post-procedure. Cholangitis was defined as fever of >38ºC with abdominal pain for more than 24 hours.^10^ Perforation was defined as the presence of free air or contrast leakage seen radiogarphically.^9^ ERCP complexity level was defined using the classification provided by Cotton PB.^11^

### Outcomes

Primary outcomes for our study included technical success and the occurrence of complications. Secondary outcomes included total procedure time, time to deep cannulation and goal attainment, inadvertent PD cannulation, PD stenting and number of attempts at cannulation.

### Statistical analysis

Sample size was calculated through OpenEpi sample size calculator with 80% power by using the postulation that PEP incidence is five to 20% with ERCP.^12^ Qualitative data was represented as frequencies and analysed using Chi square test. Quantitative data was analysed using mean±SD and Student T or Mann-Whitney U Test (non-normal). The relation between outcome measures and position of the patients was seen through univariate and logistic regression analyses. All data was analysed using SPSS V.21 with p value <0.05 considered significant.

## RESULTS

**Fig.1:**
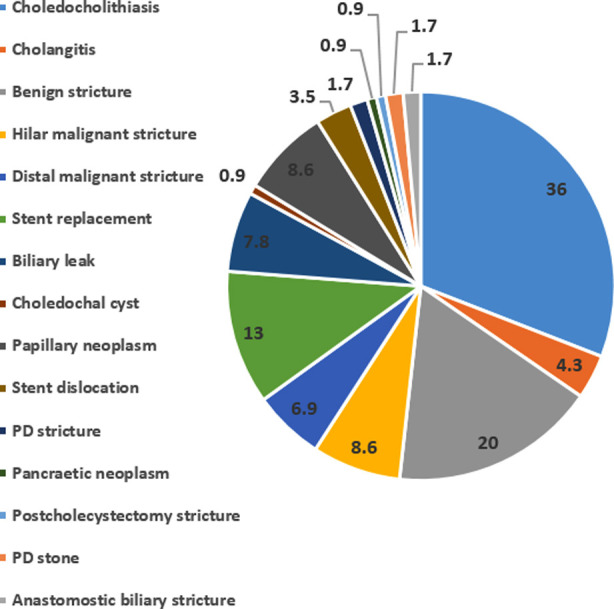
Indications

A total of 114 patients were enrolled according to the inclusion criteria with 62(54%) males and majority of the patients (42%) belonging to the age group 31-45 years. The patients who were assigned PP were relatively young with less comorbidities and a lower ASA grade (Table-I), as already explained in the methods section. The most common ERCP indication was choledocholithiasis (36%) followed by benign CBD strictures (20%), stent replacement (13%) and hilar malignant strictures (8.6%) (Fig.1).

**Table-I:** Demographics of the study cohort (n=114).

Variables	Left Lateral decubitus (n=50)	Prone (n=64)	P value
***Age (years)***			≤0.001
15-30	4(3.5)	10(8.8)	
31-45	17(15)	31(27)	
46-60	7(6)	18(16)	
61-75	15(13)	3(2.6)	
>75	7(6)	2(1.7)	
Male Gender	32(28)	30(26)	0.07
***Comorbidities***			
None	20(17.5)	37(32.5)	0.06
Diabetes mellitus	19(16.7)	6(5.5)	
Hypertension	20(17.5)	15(13)	
COPD	5(4.4)	3(2.6)	
CKD	2(1.8)	0	
CLD	2(1.8)	0	
Obesity	3(2.6)	0	
Post liver transplant	1(0.9)	1(0.9)	
Ulcerative colitis	0	1(0.9)
***ASA***			
I	21(18.4)	48(42)	0.001
II	17(15)	13(11.4)	
III	12(10.5)	3(2.6)
***Classification of papilla***			
Regular	33(29)	41(36)	0.28
Protruding	15(13)	14(12)	
Peri/intra diverticular	1(0.9)	6(5.3)	
Surgically altered	1(0.9)	3(2.6)
***Laboratory parameters***			
TLC	9.8±2.6	8.9±2.4	0.103
ALT	104±56	213±1002	0.035
AST	113±59	96±70	0.019
Bilirubin	63.5±40	49±37	0.023
Alkaline Phosphatase	373±197	285±156	0.018
GGT	173±82	138±75	0.553*
***Amylase***			
-At 6 hours	129±109	176±187	0.211
-At 24 hours	160±263	207±307	0.426

* Student T-test.

Technical success was achieved in 109(96%) patients with no statistically significant difference between the two groups through different methods used for deep biliary cannulation (Table-III). Three patients, however, had to be shifted from LL to PP for deep cannulation, two of which failed and had to be referred for percutaneous trans-hepatic biliary drainage (PTBD). The total time of procedure, localization of papilla, time for deep cannulation, time for acquiring therapeutic goal (except for using standard cannulation method, which was shorter for PP) and ERCP complexity level were all similar between the two groups.

The rate of inadvertent PD cannulation and PEP were relatively higher for the PP group but were not statistically significant through univariate and logistic regression analyses (Table-II, III). Mortality was zero for both the groups with meticulous monitoring of those patients that did develop complications. Using univariate and logistic regression analyses, the only outcome measure that showed significance was multiple cannulations in the PP group (Table-III).

**Table-II:** Outcome of ERCP in the study population (n (%) or mean±SD).

Outcome measures	Left Lateral decubitus	Prone	P value
Total time of procedure (sec)	1585±641	1578±664	0.92
Time for localization of papilla (sec)	31±17	37±41	0.811
***Time for deep cannulation (sec)***			
Standard cannulation	298±446	215±371	0.75
Double guidewire and/or pancreatic stent assisted	85±347	134±366	0.287
NKS	136±388	273±479	0.101
TPS and/or combined sphincterotomy	58±261	93±383	0.929
***Time for acquiring goal (sec)***			
Standard cannulation	961±816	545±630	0.005
Double guidewire and/or pancreatic stent assisted	154±635	225±626	0.285
NKS	257±601	518±890	0.150
TPS and/or combined sphincterotomy	118±479	141±574	0.956
PD cannulation	9(8)	20(17.5)	0.097
PD stenting	3(2.6)	8(7)	0.243
***Technical success***			
Standard cannulation	34(30)	30(26)	0.178
Double guidewire and/or pancreatic stent assisted	3(2.6)	9(8)	
Early NKS	8(7)	14(12)	
NKS as last resort	0	4(3.5)	
TPS and/or combined sphicterotomy	3(2.6)	4(3.5)	
Failed procedure	2(1.8)	3(2.6)	
***Number of attempts at cannulation***			
5	49(43)	56(49)	0.04
More than 5	1(0.9)	8(7)	
***ERCP complexity level***			
Grade 1	3(2.6)	7(6)	0.257
Grade 2	17(15)	30(26.3)	
Grade 3	28(24.6)	24(21)	
Grade 4	2(1.8)	3(2.6)	
***Complications***			
None	45(39.5)	58(51)	0.184
Cholangitis	0	2(1.8)	
Bleeding	2(1.8)	0	
Perforation	0	1(0.9)	
Pancreatitis	1(0.9)	3(2.6)	
Desaturation	2(1.8)	0	

**Table-III:** Relation of primary and secondary outcome measures with the position of the patients.

Outcome measures	Univariate analysis	Logistic regression
Multiple cannulations	0.039	0.037
Procedure failure	0.860	0.845
Inadvertent PD cannulation	0.09	0.097
ERCP complexity level	0.262	0.106
PEP	0.443	0.430

## DISCUSSION

Large volumes of research are being carried out regarding safety and efficacy of ERCP as advance GI procedures are becoming available in secondary and tertiary care facilities. There is a paucity of literature regarding the effects of position on the outcomes of ERCP in terms of operator and patient perspective.^13^ Data from the last decade shows a few small single-centre studies comparing prone to supine position, with no significant differences in success rate but greater procedural difficulty.^14^ As discussed earlier, PP is not ideal in pregnant patients, patients with rheumatological conditions and situations where there is a high intra-abdominal pressure.

LL position can be safely used in these cases and is theoretically safer, convenient and acceptable to the operators as well as the anaesthetists.^13^ The only drawback of LL position is the inferior opacification of the biliary tree and difficulty in finding the ampullary location.^13^ However, a recent RCT showed that LL is non-inferior to PP in terms of technical and clinical success rates, complication rate but showed a higher PD cannulation incident.^15^

Our study showed an equal technical and clinical success rate for both the groups (96%). The failure rate was also non-significant between the groups. Similar studies comparing patient position during ERCP concluded a technical success rate of 70 - 90% for SP, 90 – 100% for PP and 90 - 96% for LL.^16^,^17^ The total time of procedure, time for deep cannulation and time for acquiring therapeutic goal were all similar between the two groups for our study, showing non-inferiority of LL in comparison to PP. This is in contrast to a pioneer study by Park et al which reported a relatively longer cannulation time for the PP group.^9^

The only difference seen in our study was a longer procedure time to attain therapeutic goal in LL group for standard cannulation. The probable reason for this difference is the fact that more complex procedures were done in LL rather than PP cohort. However, the risk of inadvertent PD cannulation (which is an indirect marker for a difficult cannulation^13^) was lower and insignificant between the groups in contrast to a recent RCT.^15^

The rate of complications for our study was also comparable to the international data and there was non-significant difference for occurrence of complications between the two groups. All the patients were treated conservatively as in-patient and only one patient with PEP required EUS guided cystgastrostomy. This suggests that LL does not increase the risk of complications (specifically PEP) although this position entails fluoroscopic challenge due to overlapping ducts.^13^

LL position is preferred by some endoscopists especially while performing ERCP in non-intubated patients and without the help of anesthesiologist.^18^ Low rate of aspiration, better access to the mouth and airways (in case of cardio-pulmonary complications) and minimal compression effect on inferior vena cava and mesenteric veins are some of the plus points that make LL a reliable position for many endoscopy suites.^19^-^21^ However, a higher radiation exposure in LL advocates carrying out large multi-centre studies.^12^

### Limitations

It includes a single-centre setup, non-matched sample, unequal allocation randomization and the lack of data regarding degree of duct opacification in the two positions. The strengths of the study include removal of operator bias, presence of C-arm fluoroscope, extensive data collection and meticulous follow up of patients who developed complications.

## CONCLUSION

The study concludes that PP and LL positions have comparable outcomes with non-significant differences in terms of technical success rate, complications (specifically PEP), total procedure time, time required for deep cannulation and attainment of goal, ERCP complexity level and inadvertent PD cannulation. Multi-centre randomized control trials are however required locally to establish the efficacy, safety and non-inferiority of both LL and PP positions for ERCP.

### Authors’ Contribution:

**LA** contributed to the study design, pro forma, statistical analysis and drafting of the manuscript. She is responsible and accountable for the accuracy and integrity of the work

**RSAK** contributed to the idea, data collection, procedure, patient care and critical review

**FS** contributed to patient care and critical review.

**FS** contributed to data collection.

**RZAK** contributed to literature review
